# Effect of dietary arginine to lysine ratios on productive performance, meat quality, plasma and muscle metabolomics profile in fast-growing broiler chickens

**DOI:** 10.1186/s40104-018-0294-5

**Published:** 2018-11-08

**Authors:** Marco Zampiga, Luca Laghi, Massimiliano Petracci, Chenglin Zhu, Adele Meluzzi, Sami Dridi, Federico Sirri

**Affiliations:** 1 Department of Agricultural and Food Sciences, Alma Mater Studiorum - University of Bologna, Via del Florio, 2, 40064 Ozzano dell’Emilia, Italy; 2 Center of Excellence for Poultry Science, University of Arkansas Fayetteville, Fayetteville, AR 72701 USA

**Keywords:** Arginine, Broiler chicken, ^1^H–NMR spectroscopy, Meat quality, Metabolomics, Nuclear magnetic resonance, Nutrition, Productive performance

## Abstract

**Background:**

Due to the important functions of arginine in poultry, it should be questioned whether the currently adopted dietary Arg:Lys ratios are sufficient to meet the modern broiler requirement in arginine. The present study aimed, therefore, to evaluate the effects of the dietary supplementation of *L*-arginine in a commercial broiler diet on productive performance, breast meat quality attributes, incidence and severity of breast muscle myopathies and foot pad dermatitis (FPD), and plasma and muscle metabolomics profile in fast-growing broilers.

**Results:**

A total of 1,170 1-day-old Ross 308 male chicks was divided into two experimental groups of 9 replicates each fed either a commercial basal diet (CON, digestible Arg:Lys ratio of 1.05, 1.05, 1.06 and 1.07 in each feeding phase, respectively) or the same basal diet supplemented on-top with crystalline *L*-arginine (ARG, digestible Arg:Lys ratio of 1.15, 1.15, 1.16 and 1.17, respectively). Productive parameters were determined at the end of each feeding phase (12, 22, 33, 43 d). At slaughter (43 d), incidence and severity of FPD and breast myopathies were assessed, while plasma and breast muscle samples were collected and analyzed by proton nuclear magnetic resonance-spectroscopy. The dietary supplementation of arginine significantly reduced cumulative feed conversion ratio compared to the control diet at 12 d (1.352 vs. 1.401, *P* < 0.05), 22 d (1.398 vs. 1.420; *P* < 0.01) and 33 d (1.494 vs. 1.524; *P* < 0.05), and also tended to improve it in the overall period of trial (1.646 vs. 1.675; *P* = 0.09). Body weight was significantly increased in ARG compared to CON group at 33 d (1,884 vs. 1,829 g; *P* < 0.05). No significant effect was observed on meat quality attributes, breast myopathies and FPD occurrence. ARG birds showed significantly higher plasma concentration of arginine and leucine, and lower of acetoacetate, glutamate, adenosine and proline. Arginine and acetate concentrations were higher, whereas acetone and inosine levels were lower in the breast of ARG birds (*P* < 0.05).

**Conclusions:**

Taken together, these data showed that increased digestible Arg:Lys ratio had positive effects on feed efficiency in broiler chickens probably via modulation of metabolites that play key roles in energy and protein metabolism.

## Background

The formulation of diets with an adequate amino acid profile is a critical step to better exploit the genetic potential of modern broiler chickens which are characterized by rapid growth rate. Furthermore, amino acid nutrition plays a central role in animal health, welfare, and quality of poultry products, as well as in the environmental impact and sustainability of the poultry industry [1]. Current modern broilers have been selected for decades for an increased feed efficiency and enhanced breast meat yield [2]. This selection process has exerted marked changes in their body composition and, hence, in their nutritional requirements, with special regards to those of amino acids and proteins. For instance, current commercial broiler diets contain higher lysine levels than those recommended by the National Research Council (NRC) [3] to support the extraordinary breast muscle development of modern genetic lines. Dozier et al. [4] showed that lysine concentration in commercial broiler diets tended to increase from 2001 to 2005. Similarly, current recommendations [5, 6] regarding the optimal amino acid levels in broiler diets indicate higher lysine concentrations than those reported by the NRC [3]. However, when the dietary concentration of a specific amino acid is subjected to variation, the concentration of the other amino acids should be modified accordingly to maintain the ideal amino acid profile. Indeed, as suggested by Kidd et al. [7], increasing the dietary concentration of lysine without considering other important amino acids such as threonine or arginine may lead to a marginal deficiency of those.

Arginine plays crucial roles in different metabolic, pathophysiological and immunological processes in poultry as elegantly reviewed by Fernandes and Murakami [8], Khajali and Wideman [9] and Fouad et al. [10]. Due to the lack of a functional urea cycle [11], broilers are not able to synthesize endogenous *L*-arginine and therefore it is considered as an essential amino acid. Indeed, chickens exclusively rely on the dietary sources of arginine and hence a proper amount of it should be provided with the diet. The specific relationship between dietary arginine and lysine has been defined, and any deficiency or excess could have negative effects on plasma and muscle amino acid concentrations and thereby growth performances [12]. This effect is more marked with an excess of lysine (low Arg:Lys ratio) rather than an excess of arginine (high Arg:Lys ratio) [12]. The excess of dietary lysine was reported not to affect the digestibility or absorption of arginine but mainly to inhibit renal reabsorption and to stimulate kidney arginase activity [12]. According to the NRC [3], the optimal Arg:Lys ratio should be 1.14 during the first 3 weeks, 1.10 from 3 to 6 weeks, and 1.18 from 6 to 8 weeks. On the other hand, Baker [13] indicated lower Arg:Lys ratio than the NRC [3] (1.05, 1.08 and 1.08 in 0-3, 3–6 and 6–8 weeks of broiler age, respectively). Balnave and Brake [12] suggested that, based on referenced literature, the optimum Arg:Lys ratio should range from 0.90 to 1.18. More recent reports suggested a ratio of 1.05, 1.08 and 1.08 in 0-21 d, 21-42 d, and 42-56 d, respectively [14]. Similarly, other current nutrition specifications [5, 6] indicated lower Arg:Lys ratios than the NRC [3]. In addition, the composition of amino acids in the whole-body of 10-day-old chicks showed that arginine content was 111% in respect to lysine [14]. In commercial diets, especially when the use of animal by-products is not allowed either by the legislation (e.g. European countries) or by voluntary decision (e.g. vegetable-only diet), the Arg:Lys levels are usually lower than the requirements reported by the NRC [3]. However, considering the important functions of arginine described above, it should be questioned whether the status quo of dietary Arg:Lys ratios are sufficient to meet the modern broiler requirement. Deficiencies or excess of arginine may have a strong adverse effect on animal health, welfare and productivity as well as on the economic and environmental sustainability of the poultry industry. There is also a paucity of scientific information regarding the effects of different Arg:Lys ratios in broiler diets on meat quality attributes and occurrence of breast muscle myopathies. *L*-arginine can be converted into citrulline and nitric oxide by the enzyme nitric oxide synthase [8, 9]. Nitric oxide has shown marked vasodilator properties [9] and it could enhance blood flow to the breast muscle alleviating the hypoxic condition usually observed in breasts affected by severe woody breast (WB) or white striping (WS) myopathies [15, 16]. Very recently, Bodle et al. [17] showed that WB, but not WS, average score was significantly reduced by increasing the level of dietary arginine.

The extraordinary advances achieved in innovative analytical techniques such as nuclear magnetic resonance (NMR) pave the possibility to investigate the global variations of metabolite profiles in body fluids or tissues in response to dietary treatments [18]. Therefore, this study was carried out to determine the effects of dietary arginine supplementation on growth performance, breast meat quality, incidence and severity of breast muscle myopathies and foot pad dermatitis, as well as plasma and muscle metabolomics profile in modern broilers.

## Methods

### Animals and housing

A total of 1,170 one-day-old Ross 308 male chicks, obtained from the same breeder flock and incubated in the same environmental conditions, was vaccinated (coccidiosis, infectious bronchitis virus, Marek’s disease virus, Newcastle and Gumboro disease) and allotted to an environmental controlled poultry house. Chicks were divided in 18 pens of 6 m^2^ each (9 replications/group, 65 birds/replication, 11 birds/m^2^) and chopped straw (2 kg/m^2^) was used as litter material. Replications were distributed in randomized blocks inside the poultry house in order to limit any environmental effect. Stocking density was defined according to the legislation in force (maximum 33 kg/m^2^) [19]. Two circular pan feeders able to guarantee at last 2 cm of front space/bird and 10 nipples were provided for each pen. A photoperiod of 23L:1D of artificial light was adopted in the first 7 d and in the last 3 d of trial, while 18L:6D was used for the remaining days [19]. The environmental temperature was settled according to the age of the birds following the management guide provided by the breeding company. Birds were handled, raised and processed according to the European legislation [19–21]. The experiment was approved by the Ethical Committee of the University of Bologna (ID: 928/2018).

### Experimental diets

The same commercial corn-wheat-soybean basal diet (Table 1) was used to produce both the experimental diets. The basal diet was formulated to meet or slightly exceed the Ross 308 nutrition recommendations [5] and maintaining the ideal amino acid profile. The feeding program was composed of 4 phases: starter (0–12 d), grower I (13–22 d), grower II (23–33 d) and finisher (34–43 d). The CON group received the basal diet without any arginine supplementation (digestible Arg:Lys ratio = 1.05, 1.05, 1.06, 1.07 in starter, grower I, grower II and finisher phase, respectively). The ARG diet was obtained by supplementing on-top the basal diet with 1.20, 1.15, 1.10 and 0.95 g/kg of crystalline *L*-arginine (purity 99%, Barentz, Hoofddorp, The Netherlands) in starter, grower I, grower II and finisher feeding phase, respectively. Samples were obtained from both the experimental diets to evaluate proximate composition. Moisture and ash content were determined in duplicate according to the Association of Official Analytical Chemists procedure [22]. Crude protein content was assessed by the standard Kjeldahl copper catalyst method as reported in AOAC [22]. Crude fat was determined using the Soxhlet method [22], which allowed to extract the ethyl-ether soluble substances contained in the sample. The amino acid concentration of the experimental diets was analyzed by AMINOLab® (Evonik Industries, Hanau, Germany). Digestible amino acid values were calculated by multiplying digestibility coefficients [23] to the analyzed total amino acid content of each ingredient. In the ARG diet, the crude protein concentration was 23.4, 22.7, 20.3 and 18.2% in starter, grower I, grower II and finisher feeding phase, respectively. The calculated digestible lysine and arginine concentration was 1.25, 1.15, 1.05 and 0.93% and 1.44, 1.32, 1.22 and 1.09%, corresponding to digestible Arg:Lys ratios of 1.15, 1.15, 1.16 and 1.17, respectively. The values concerning the CON diet are reported in Table 1. Both the diets were administered in a mash form and feed and water provided for ad libitum consumption.

**Table 1:** Composition of the basal diet in each feeding phase

Items	0–12 d	13–22 d	23–33 d	34–43 d
Ingredients, g/100 g				
Corn	33.4	36.7	19.2	15.0
White corn	0.00	0.00	15.0	18.1
Wheat	20.0	20.0	25.0	30.0
Vegetable oil	2.45	2.68	3.61	3.97
Soybean meal 48%	18.2	20.2	14.2	9.33
Full-fat soybean	10.0	10.0	15.0	15.0
Concentrated SBM	5.00	0.00	0.00	0.00
Corn gluten	2.00	2.00	0.00	0.00
Pea	3.00	3.00	3.00	3.00
Sunflower	2.00	2.00	2.00	3.00
Lysine sulphate	0.54	0.53	0.46	0.43
*DL*-Methionine	0.29	0.00	0.00	0.00
Methionine hydroxy analogue	0.00	0.32	0.33	0.26
*L*-Threonine	0.12	0.11	0.10	0.08
Choline chloride	0.10	0.10	0.05	0.00
Calcium carbonate	0.53	0.52	0.60	0.69
Dicalcium phosphate	1.29	0.80	0.47	0.21
Sodium chloride	0.29	0.30	0.23	0.21
Sodium bicarbonate	0.05	0.05	0.15	0.25
Premix vit.-min.^1^	0.54	0.46	0.38	0.30
Phytase	0.05	0.05	0.05	0.05
Xylanase	0.05	0.05	0.05	0.05
Emulsifier	0.08	0.08	0.08	0.08
Proximate composition				
AME, MJ/kg	13.0	13.2	13.7	13.9
Dry matter^a^, %	88.8	88.2	88.5	88.5
Crude protein^a^, %	23.2	22.8	19.8	18.2
Total lipid^a^, %	6.25	6.51	8.29	8.64
Crude fiber, %	2.96	2.92	2.99	3.08
Ash^a^, %	5.24	4.60	4.29	4.03
Ca (total), %	0.77	0.62	0.55	0.50
P (total), %	0.61	0.51	0.44	0.38
Dig. Lysine^a^, %	1.25	1.15	1.05	0.94
Dig. Arginine^a^, %	1.32	1.21	1.11	1.00
Dig. Met. + Cys^a^, %	0.93	0.85	0.79	0.70
Dig. Threonine^a^, %	0.81	0.75	0.68	0.61
Dig. Valine^a^, %	0.94	0.87	0.79	0.72
Dig. Isoleucine^a^, %	0.84	0.77	0.70	0.63
Dig. Arg:Lys	1.05	1.05	1.06	1.07
Dig. Lys:AME, g/MJ	0.96	0.87	0.77	0.68

^1^ Provided the following per kg of starter diet: vitamin A (retinyl acetate), 13,000 IU; vitamin D_3_ (cholecalciferol), 4000 IU; vitamin E (*DL*-α-tocopheryl acetate), 80 IU; vitamin K (menadione sodium bisulfite), 3 mg; riboflavin, 6.0 mg; pantothenic acid, 6.0 mg; niacin, 20 mg; pyridoxine, 2 mg; folic acid, 0.5 mg; biotin, 0.10 mg; thiamine, 2.5 mg; vitamin B_12_ 20 μg; Mn, 100 mg; Zn, 85 mg; Fe, 30 mg; Cu, 10 mg; I, 1.5 mg; Se, 0.2 mg; ethoxyquin, 100 mg

^a^Analysed values. Amino acid concentration of the experimental diets was analyzed by AMINOLab® (Evonik Industries, Hanau, Germany). Digestible amino acid (dig.) values were calculated by multiplying digestibility coefficients [23] to the analyzed total amino acid content of each ingredient

### Productive performance and slaughtering measurements

Number and weight of the birds were recorded on a pen basis at housing (0 d), at each diet switch (12, 22, 33 d) and at slaughter (43 d). Feed intake was recorded at the end of each feeding phase (12, 22, 33, 43 d). Mortality was recorded on a daily basis and dead birds were weighed, necropsied, and recorded to calculate the mortality percentage and to correct the productive performance results. Body weight (BW), daily weight gain (DWG), daily feed intake (DFI) and feed conversion ratio (FCR) and cumulative FCR were determined for each feeding phase and for the overall rearing period. At 43 d, all birds were processed in a commercial plant and slaughtered according to the legislation in force using water-bath electrical stunning (200–220 mA, 1,500 Hz). Birds and carcasses belonging to the different experimental groups were clearly identified and kept separated throughout the processing phases. For each experimental group, all the birds were mechanically processed and eviscerated to obtain carcass yield on a group basis by removing blood, feathers, head, neck, viscera, abdominal fat, and feet. The overall carcass weight of each group was recorded after air-chilling and carcass yield calculated as percentage of body liveweight.

Similarly, skinless and deboned breast was mechanically obtained from the carcass and yield calculated on a group basis as percentage of carcass weight. The incidence and severity of foot pad dermatitis (FPD) were macroscopically evaluated on all birds (1 ft/bird) using the 3-points scale evaluation system proposed by Ekstrand et al. [24] [score 0 = no lesions; score 1 = mild lesions (< 0.8 cm); score 2 = severe lesions (> 0.8 cm)].

### Blood and breast muscle collection

At slaughter (43 d), 9 birds/group (1 bird/replication) selected with similar BW and clearly labelled were subjected to blood withdrawal. Blood was obtained from the wing vein, collected into 4 mL lithium-heparin vials and immediately centrifuged (4,000×*g* for 15 min) to obtain plasma, which was transferred into 1.5 mL labeled vials and stored at − 80 °C until metabolomic analysis. From the same 9 birds/group, a sample of *Pectoralis major* muscle was obtained, put into a 1.5-mL vial, immediately frozen under liquid nitrogen and then kept at − 80 °C until metabolomic analysis. The samples were consistently obtained from the same area of the breast muscle showing no macroscopic defects.

### Incidence of breast meat abnormalities

The incidence and severity of white striping (WS), wooden breast (WB) and spaghetti meat abnormalities (SM) were evaluated on 150 randomly collected breasts/group approximately 24 h after processing. For each defect, a 3 points-scale evaluation system (NOR: normal; MOD: moderate; SEV: severe) was used to classify the magnitude of the myopathy. All the scorings were performed by the same operator in the same environmental conditions. For WS, the classification criteria were the dimension of white striation [25], whereas the hardness at palpation was used for the WB defect [26]. Finally, the proneness to show muscle deconstruction in response to an external stimulus (finger pinching), as described by Sirri et al. [27], was used to score the breasts according to the SM defect.

### Meat quality attributes

Twelve breasts/group not showing macroscopic defects (e.g. visual signs of muscle myopathies, hemorrhages or lesions) and obtained from carcasses with BW similar to the average BW of each group were collected and used to assess meat quality attributes and proximate composition. Breast muscle pH was determined 48 h post-mortem using a modification of the iodoacetate method [28] as previously reported [29]. The system color profile [30] of breast muscle was obtained by a reflectance colorimeter (Minolta Chroma Meter CR-300, Minolta Italia S.p.A., Milan, Italy) using illuminant source C. The results were reported as lightness (L^*^), redness (a^*^), and yellowness (b^*^) and represent the average of 3 independent measurements performed on the medial surface of the fillet (bone side) in an area showing no evident color defects. In addition, a parallelepiped meat cut (8 cm × 4 cm × 3 cm) weighing about 80 g was excised from the cranial part of each fillet and used to determine drip (of refrigerated storage) and cooking losses (in a water bath at 80 °C for 45 min) using the same procedures described in our previous study [29]. A second parallelepiped meat cut (8 cm × 4 cm × 2 cm) weighing about 60 g was excised from the middle part of each fillet and was individually labeled and tumbled with a 15% (*wt/wt*) brine solution containing sodium tripolyphosphate (2.3%) and sodium chloride (7.6%) and subsequently cooked in a water bath at 80 °C for 25 min. Marinade uptake and cooking losses were calculated for each sample [29].

Proximate analysis was performed on breast meat samples to assess moisture, crude protein, total fat and ash content. Moisture and ash were obtained in duplicate according to the procedure described by the Association of Official Analytical Chemists [22]. Total fat and crude protein content were determined using the chloroform:methanol extraction procedure reported by Folch et al. [31] and the standard Kjeldahl copper catalyst method [22], respectively.

### Plasma and muscle metabolomics analysis

Plasma samples were prepared for proton NMR (^1^H-NMR) analysis by centrifuging 650 μL of each sample for 15 min at 15,000 r/min (18,630 × *g*) and 4 °C. 500 μL of supernatant were added to 100 μL of a D_2_O solution of 2,2,3,3-D4-3-(trimethylsilyl)-propionic acid sodium salt 10 mmol/L, used as NMR chemical-shift reference, buffered at pH 7.00 by means of 1 mol/L phosphate buffer. Finally, each sample was centrifuged again at the above conditions.

Meat samples were prepared for NMR analysis by adding 0.5 g of meat to 3 mL distilled water and by homogenizing the mixture for 2 min by means of a high-speed disperser (IKA, USA). One mL of the obtained sample was centrifuged for 15 min at 15,000 r/min (18,630 × *g*) and 4 °C. To remove fat from samples, 700 μL of supernatant were added to 800 μL CHCl_3_, vortex mixed for 3 min and centrifuged again at the above conditions. 500 μL of supernatant were added to 200 μL of a D_2_O solution of 2,2,3,3-D4-3-(trimethylsilyl)-propionic acid sodium salt 10 mmol/L, used as NMR chemical-shift reference, buffered at pH 7.00 ± 0.02 by means of 1 mol/L phosphate buffer. 10 μL of NaN_3_ 2 mmol/L were also added to avoid microbial proliferation. Finally, each sample was centrifuged again at the above conditions.

^1^H-NMR spectra were recorded at 298 K with an AVANCE III spectrometer (Bruker, Milan, Italy) operating at a frequency of 600.13 MHz. Following Ventrella et al. [32], the signals from broad resonances originating from large molecules were suppressed by a CPMG-filter composed by 400 echoes with a *τ* of 400 μs and a 180° pulse of 24 μs, for a total filter of 330 ms. The water residual signal was suppressed by means of presaturation. This was done by employing the cpmgpr1d sequence, part of the standard pulse sequence library. Each spectrum was acquired by summing up 256 transients using 32,000 data points over a 7184 Hz spectral window, with an acquisition time of 2.28 s. In order to apply NMR as a quantitative technique [33], the recycle delay was set to 5 s, keeping into consideration the relaxation time of the protons under investigation. ^1^H-NMR spectra baseline-adjusted by means of the peak detection according to the “rolling ball” principle [34] implemented in the baseline R package [35]. In order to make the points pertaining to the baseline randomly spread around zero, a linear correction was then applied to each spectrum. Differences in water and fibers content among samples were taken into consideration by probabilistic quotient normalization [36] applied to the entire spectra array. The signals were assigned by comparing their chemical shift and multiplicity with the Human Metabolome Database [37] and Chenomx software library (Chenomx Inc., Canada, ver. 10). This was done by taking advantage of the “autofit” utility of Chenomx software (ver. 8.3).

### Statistical analysis

Once assessed that the effect of the block as well as the interaction between block and dietary treatments were not significant, block effect was not considered in the analysis and productive performance data were analyzed applying the Student *t*-test [38], considering the dietary supplementation of *L*-arginine as independent variable. Pen was considered as the experimental unit for productive performance data. Prior to analysis, mortality data were submitted to arcsine transformation. Similarly, meat quality attributes were analyzed by means of the Student *t*-test [38], considering the bird as experimental unit. The occurrence of FPD and breast meat abnormalities was analyzed using the Chi-square test considering the bird as experimental unit. Differences were considered statistically significant when *P*-value was lower 0.05.

Regarding metabolomics, molecules whose concentration varied in relation to the dietary supplementation of *L*-arginine were compared by means of Wilcoxon test in agreement with previous investigations [32, 39]. For this purpose, a significance limit *P*-value of 0.05 was accepted. To highlight the underlying trends characterizing the samples, principal component analysis model in its robust version (rPCA) was built on the molecules concentrations, centered and scaled to unity variance, according to Hubert [40]. For each rPCA model, the scoreplot, that is the projection of the samples in the PC space, tailored to highlight the underlying structure of the data, was calculated. Besides, the correlation plot was obtained by relating the concentration of each variable to the components of the rPCA model, therefore tailored to highlight the most important molecules in determining the trends highlighted by the scoreplot.

## Results

### Productive performance and slaughtering measurements

The productive performance results are reported in Table 2. Both the experimental groups showed similar body weight at the beginning of the trial. After 12 d, ARG group showed a lower FCR compared to CON group (1.352 vs. 1.401, *P* < 0.05) whereas BW, DWG, and DFI remained unaffected between both groups. At 22 d, cumulative FCR was significantly lower in ARG compared to the control group (1.398 vs. 1.420, *P* < 0.01). Dietary supplementation did not elicit any significant effect on the other productive traits. After 33 d, ARG-fed birds exhibited higher BW (1,884 vs. 1,829 g, *P* < 0.05) and lower cumulative FCR (1.494 vs. 1.524, *P* < 0.05) compared to the CON-fed group. Furthermore, DWG tended to be higher and FCR tended to be lower in ARG compared to CON group (93.1 vs. 89.3 g/(bird·d), and 1.571 vs. 1.610, *P* = 0.08). In the finisher feeding phase (34-43 d), no significant difference was observed between the experimental groups. In the overall period of trial (0–43 d), the dietary supplementation of arginine tended to improve FCR (1.646 vs. 1.675, respectively for ARG and CON; *P* = 0.09), while it had only limited effect on BW, DWG and DFI. Mortality rate was not significantly affected by the dietary treatment in each feeding phase as well as in the overall period of trial.

**Table 2:** Productive performance of broiler chickens fed a commercial basal diet (CON, *n* = 9; digestible Arg:Lys = 1.05, 1.05, 1.06 and 1.07) or the same basal diet supplemented with *L*-arginine (ARG, *n* = 9; digestible Arg:Lys = 1.15, 1.15, 1.16 and 1.17)

Variables	CON	ARG	SEM	*P*-value
0–12 d				
Chick body weight, g/bird	37.1	36.9	0.09	0.27
Body weight, g/bird	288.5	293.0	2.47	0.38
Daily weight gain, g/(bird·d)^a^	21.0	21.3	0.20	0.39
Daily feed intake, g/(bird·d)^a^	29.3	28.8	0.19	0.21
Feed conversion rate (0–12 d)^a^	1.401	1.352	0.01	0.02
Mortality, %	0.00	0.17	0.01	0.33
13–22 d				
Body weight, g/bird	846.7	856.1	6.08	0.46
Daily weight gain, g/(bird·d)^a^	55.8	56.3	0.43	0.56
Daily feed intake, g/(bird·d)^a^	79.7	79.8	0.60	0.92
Feed conversion rate (13–22 d)^a^	1.429	1.419	0.01	0.37
Cumulative feed conversion rate (0–22 d)^a^	1.420	1.398	0.01	< 0.01
Mortality, %	0.34	0.52	0.02	0.69
23–33 d				
Body weight, g/bird	1829	1884	12.8	0.03
Daily weight gain, g/(bird·d)^a^	89.3	93.1	1.11	0.08
Daily feed intake, g/(bird·d)^a^	143.6	145.5	1.02	0.39
Feed conversion rate (23–33 d)^a^	1.610	1.571	0.01	0.09
Cumulative feed conversion rate (0–33 d)^a^	1.524	1.494	0.01	0.02
Mortality, %	0.17	0.35	0.01	0.55
34–43 d				
Body weight, g/bird	2864	2920	26.0	0.30
Daily weight gain, g/(bird·d)^a^	101.6	102.3	1.62	0.83
Daily feed intake, g/(bird·d)^a^	197.1	196.9	1.41	0.94
Feed conversion rate (34–43 d)^a^	1.949	1.926	0.02	0.62
Mortality, %	1.37	1.71	0.02	0.62
0–43 d				
Body weight, g/bird	2864	2920	26.0	0.30
Daily weight gain, g/(bird·d)^a^	65.7	67.0	0.60	0.30
Daily feed intake, g/(bird·d)^a^	109.1	109.2	0.54	0.94
Feed conversion rate (0–43 d)^a^	1.675	1.646	0.01	0.09
Mortality, %	1.88	2.74	0.02	0.21

^a^ Corrected for mortality

**Fig. 1:**
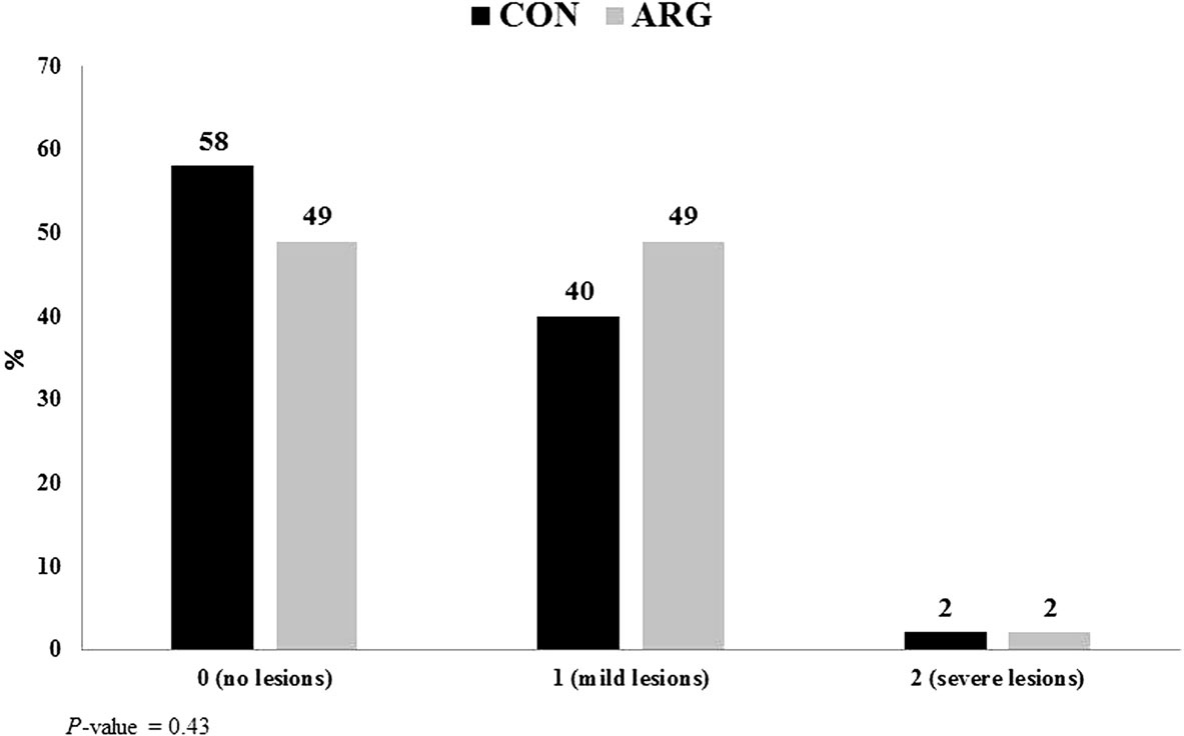
Incidence and severity of foot pad dermatitis in CON and ARG broiler chickens. CON, *n* = 574: digestible Arg:Lys = 1.05, 1.05,  1.06 and 1.07 in starter, grower I, grower II and finisher feeding phase, respectively. ARG, *n* = 569: digestible Arg:Lys = 1.15, 1.15, 1.16 and 1.17, respectively. [0 = no lesions; 1 = mild lesions (< 0.8 cm); 2 = severe lesions (> 0.8 cm)]

At processing, eviscerated carcass yield was 71.3 and 70.9% for ARG and CON, respectively. Skinless breast yield, expressed as percentage of carcass weight, was 30.4 and 29.3% for ARG and CON, respectively. As shown in Fig. 1, no significant effect of dietary arginine supplementation was detected on the incidence and severity of FPD.

### Incidence of breast muscle myopathies and meat quality

The dietary supplementation of arginine did not affect the incidence and severity of WS, WB and SM (Table 3). The results of the evaluation of the breast meat quality attributes are shown in Table 4. Breast meat pH, color, drip and cooking losses, as well as marinade uptake and purging loss, showed no significant changes in response to the dietary treatment. Considering the proximate composition of breast meat, the dietary supplementation of arginine had no significant effect on moisture, crude protein, total fat, as well as ash content (Table 4).

**Table 3:** Incidence and severity of white striping, wooden breast and spaghetti meat defect in breast muscle of broiler chickens fed a commercial basal diet (CON, *n* = 150; digestible Arg:Lys = 1.05, 1.05, 1.06 and 1.07) or the same basal diet supplemented with *L*-arginine (ARG, *n* = 150; digestible Arg:Lys = 1.15, 1.15, 1.16 and 1.17)

Variables	CON	ARG
White striping		
0 (no lesions), %	17	7
1 (mild lesions), %	52	53
2 (severe lesions), %	31	39
Chi-square	0.08	
Wooden breast		
0 (no lesions), %	43	44
1 (mild lesions), %	38	37
2 (severe lesions), %	19	19
Chi-square	0.99	
Spaghetti meat		
0 (no lesions), %	65	60
1 (mild lesions), %	29	33
2 (severe lesions), %	6	7
Chi-square	0.77

**Table 4:** Meat quality attributes and proximate composition of *Pectoralis major* muscle belonging to broiler chickens fed a commercial basal diet (CON, *n* = 12; digestible Arg:Lys = 1.05, 1.05, 1.06 and 1.07) or the same basal diet supplemented with synthetic *L*-arginine (ARG, *n* = 12; digestible Arg:Lys = 1.15, 1.15, 1.16 and 1.17)

Variables	CON	ARG	SEM	*P*-value
Meat quality attributes				
pH 48 h post-mortem	5.81	5.76	0.05	0.44
Lightness (L*)	59.5	60.4	0.94	0.29
Redness (a*)	2.07	2.07	0.25	0.75
Yellowness (b*)	5.85	6.46	0.44	0.23
Drip loss, %	1.97	1.81	0.18	0.47
Cooking loss – raw meat, %	15.4	15.4	1.02	0.98
Marinade uptake, %	10.6	9.9	0.95	0.61
Cooking loss – marinated meat, %	12.5	12.9	0.70	0.58
Proximate composition				
Moisture, %	76.4	76.8	0.49	0.20
Crude protein, %	21.7	21.8	0.43	0.91
Total fat, %	1.71	1.61	0.19	0.59
Ash, %	1.40	1.34	0.17	0.75

### Plasma and breast muscle metabolome

**Fig. 2:**
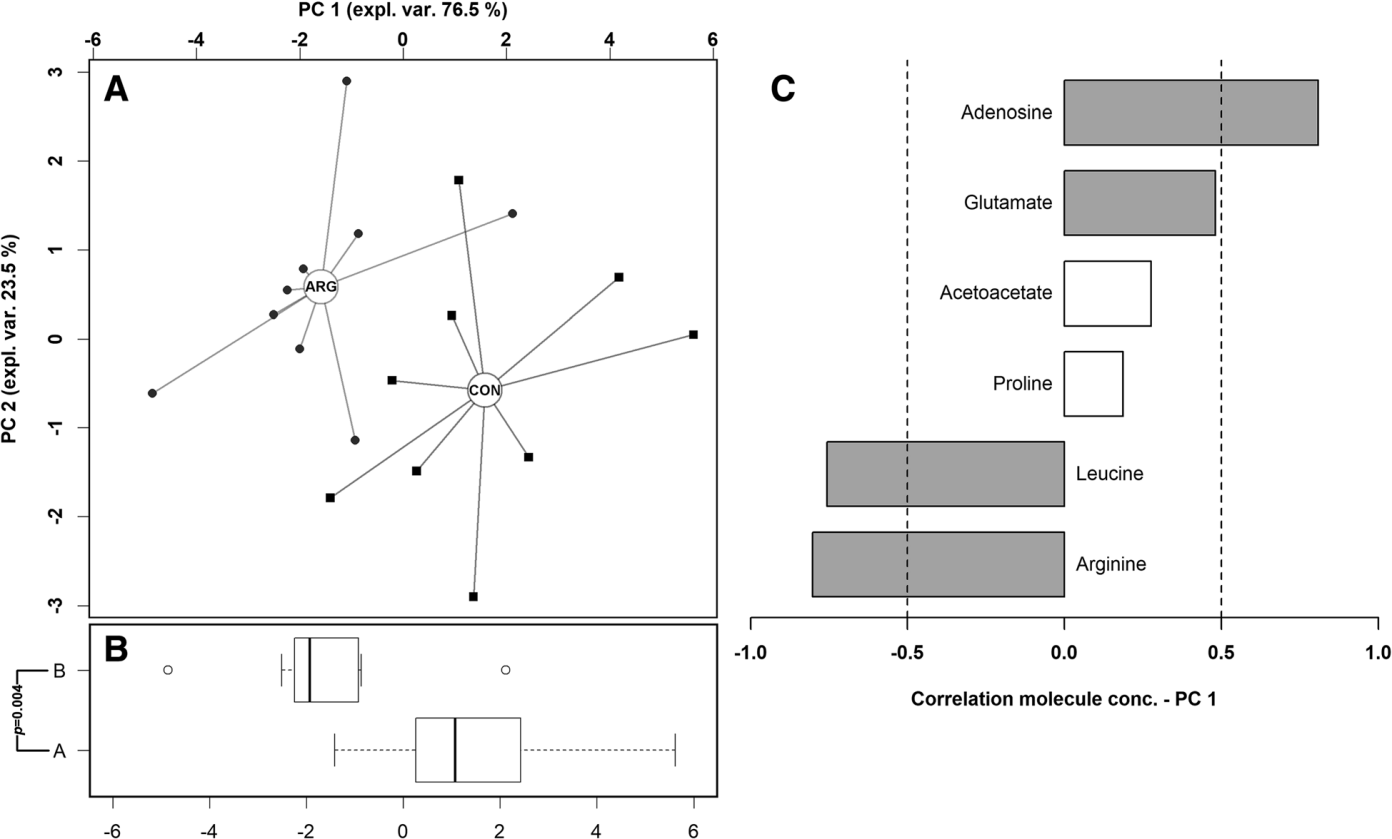
Robust Principal Component Analysis on plasma metabolites showing differential expression between CON and ARG groups. CON, *n* = 9; digestible Arg:Lys = 1.05, 1.05, 1.06 and 1.07 in starter, grower I, grower II and finisher feeding phase, respectively. ARG, *n* = 9; digestible Arg:Lys = 1.15, 1.15, 1.16 and 1.17, respectively. **a** In the scoreplot, samples from chickens fed different diets are represented with squares and circles respectively. The wide, empty circles represent the median of the samples at the various time-points. **b** Boxplot summarizing the position of the subjects along PC 1. **c** Loadingplot reports the correlation between the concentration of each substance and its importance over PC 1. Highly significant correlations (*P* < 0.05) are highlighted with gray bars

^1^H-NMR spectra were registered on plasma samples and 62 molecules quantified. Six molecules, listed in Table 5, exhibited significant variation in their plasma concentration in response to the dietary supplementation of arginine. ARG birds showed significantly higher plasma arginine and leucine concentrations, whereas plasma acetoacetate, adenosine, glutamate and proline were more abundant in CON birds. To obtain an overview about the molecules undergoing the greatest differences between the groups, the 6 molecules of Table 5 were employed as a basis for a rPCA model, shown in Fig. 2.

**Table 5:** Relative concentration of differentially expressed plasma metabolites in broiler chickens received a commercial basal diet (CON, *n* = 9; digestible Arg:Lys = 1.05, 1.05, 1.06 and 1.07) or the same basal diet supplemented with synthetic *L*-arginine (ARG, *n* = 9; digestible Arg:Lys = 1.15, 1.15, 1.16 and1.17)

Metabolites^a^	CON	ARG	Trend	*P*-value
Arginine, mmol/L	4.30 × 10^−3^ ± 6.86 × 10^− 5^	5.64 × 10^− 3^ ± 1.08 × 10^− 4^	↑	< 0.01
Leucine, mmol/L	1.79 × 10^−1^ ± 2.41 × 10^− 3^	2.11 × 10^− 1^ ± 3.93 × 10^− 3^	↑	0.01
Acetoacetate, mmol/L	5.94 × 10^− 2^ ± 1.60 × 10^− 3^	4.35 × 10^− 2^ ± 1.08 × 10^− 3^	↓	0.02
Glutamate, mmol/L	7.31 × 10^− 2^ ± 6.31 × 10^− 4^	6.56 × 10^− 2^ ± 4.96 × 10^− 4^	↓	0.01
Adenosine, mmol/L	1.38 × 10^− 3^ ± 2.39 × 10^− 4^	7.84 × 10^− 5^ ± 3.92 × 10^− 5^	↓	0.04
Proline, mmol/L	1.23 × 10^− 1^ ± 2.18 × 10^− 3^	1.05 × 10^− 1^ ± 2.06 × 10^− 3^	↓	0.04

^a^ Results are reported as mean ± SEM

**Fig. 3:**
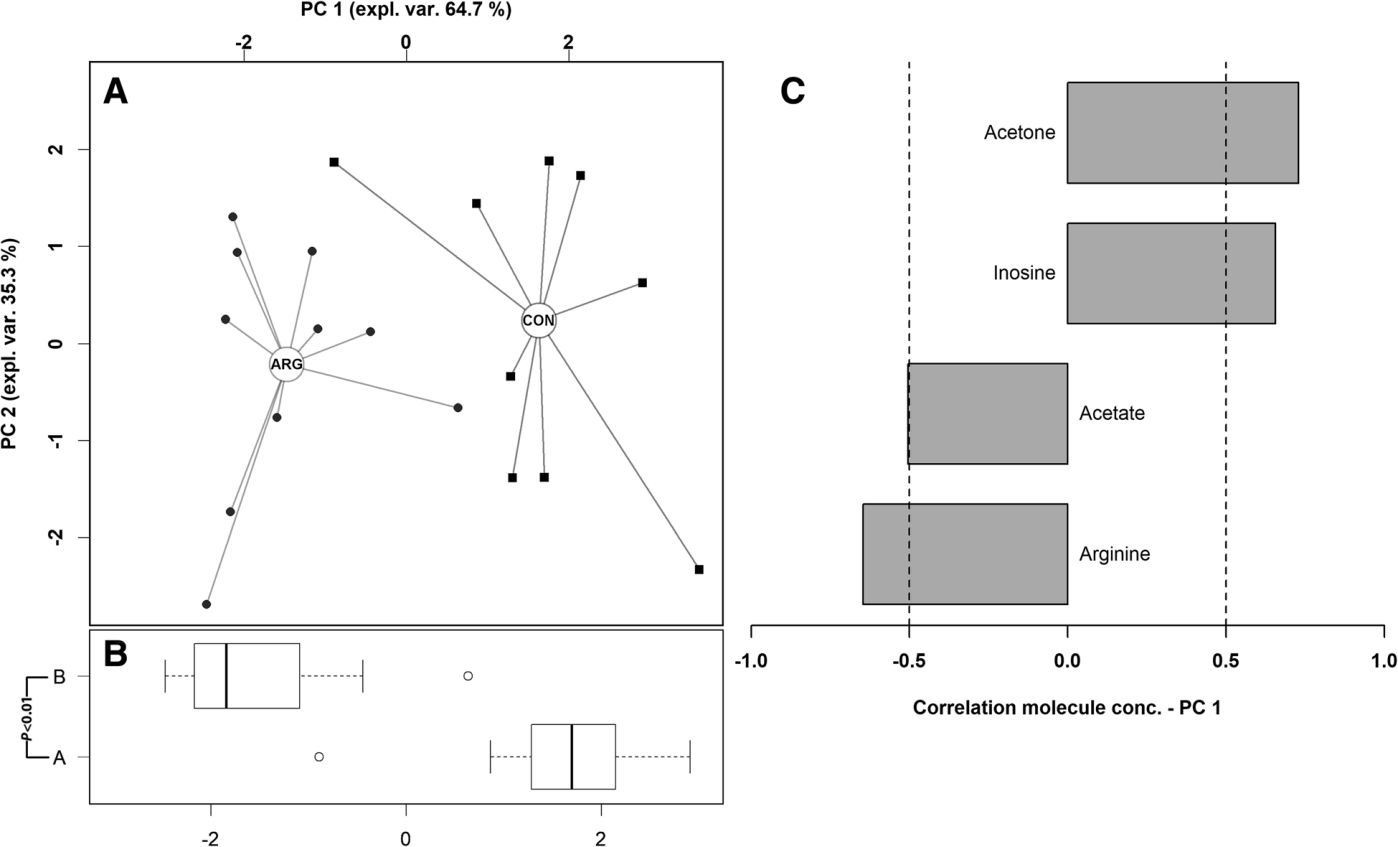
Robust Principal Component Analysis on breast metabolites showing differential expression between CON and ARG groups. CON, *n* = 9; digestible Arg:Lys = 1.05, 1.05, 1.06 and 1.07 in starter, grower I, grower II and finisher feeding phase, respectively. ARG, *n* = 9; digestible Arg:Lys = 1.15, 1.15, 1.16 and 1.17, respectively. **a** In the scoreplot, samples from chickens fed different diets are represented with squares and circles respectively. The wide, empty circles represent the median of the samples at the various time-points. **b** Boxplot summarizing the position of the subjects along PC 1. **c** Loadingplot reports the correlation between the concentration of each substance and its importance over PC 1. Highly significant correlations (*P* < 0.05) are highlighted with gray bars

In parallel to what was done on plasma, ^1^H-NMR spectra were obtained from breast muscle samples. From a total of 37 quantified molecules, 4 showed a significantly different concentration between CON and ARG group. Breast muscle from ARG group exhibited higher levels of arginine and acetate and lower levels of acetone and inosine (Table 6). The rPCA model obtained using the 4 molecules of Table 6 is shown in Fig. 3.

**Table 6:** Relative concentration of differentially expressed metabolites in breast muscle of broiler chickens fed a commercial basal diet (CON, *n* = 9; digestible Arg:Lys = 1.05, 1.05, 1.06 and 1.07) or the same basal diet supplemented with synthetic *L*-arginine (ARG, *n* = 9; digestible Arg:Lys = 1.15, 1.15, 1.16 and 1.17)

Metabolites^a^	CON	ARG	Trend	*P*-value
Arginine, mmol/L	3.35 × 10^−4^ ± 5.59 × 10^− 6^	3.98 × 10^− 4^ ± 3.78 × 10^− 6^	↑	< 0.01
Acetate, mmol/L	2.48 × 10^− 4^ ± 1.29 × 10^− 6^	2.67 × 10^− 4^ ± 2.16 × 10^− 6^	↑	0.02
Inosine, mmol/L	4.88 × 10^− 4^ ± 8.97 × 10^− 6^	4.06 × 10^− 4^ ± 3.90 × 10^− 6^	↓	< 0.01
Acetone, mmol/L	2.35 × 10^− 5^ ± 1.71 × 10^− 6^	1.10 × 10^− 5^ ± 5.86 × 10^− 7^	↓	0.03

^a^ Results are reported as mean ± SEM

## Discussion

In the present study, broilers were fed either a basal diet (CON group), formulated to meet or slightly exceed the current recommendations [5] and widely used in commercial practice, or the same basal diet supplemented with crystalline *L*-arginine (ARG group) to increase the digestible Arg:Lys ratio. Considering other published studies aimed at evaluating the effects of the dietary supplementation of arginine in broilers, huge differences regarding bird’s genotype, age, and sex, composition of the basal diet, number and length of feeding phases, and inclusion rate of arginine, were observed and therefore care should be used in comparing results from different studies [41–54].

As for productive aspects, the dietary supplementation of arginine at the level tested in this study improved cumulative FCR at 12, 22 and 33 d and tended to improve it in the overall period of trial (0–42 d). ARG birds exhibited improved FCR in each feeding phase, even though significant differences between the groups were detected only in the starter phase (0–12 d). Corzo and Kidd [41] stated that the dietary supplementation of arginine might exert positive effects during the starter phase by counteracting the early microbial challenges and aiding the immune system development. Similarly to our findings, Jahanian and Khalifeh-Gholi [42] reported that broilers fed a diet with an arginine level of 100% of NRC recommendation (total Arg:Lys = 1.14 and 1.10 in starter and grower phase, respectively) exhibited lower FCR at 21 d, as well as from 1 to 42 d, if compared to broilers receiving an arginine-deficient diet (90% NRC, Arg:Lys = 1.02 and 0.99 in starter and grower phase, respectively). It has been reported that feed efficiency was affected at 10, 24 and 46 d of age by increasing the level of digestible arginine from 100% of Ross recommendations to 153, 168 and 183% [43]. On the other hand, no significant difference was observed in terms of FCR in broilers fed diets with arginine levels either to meet (100%) or exceed (105 and 110%) the NRC recommendations [44]. Similarly, the administration of graded levels of arginine (0.45, 0.90, 1.35 and 1.90%) in an arginine-deficient diet (total Arg:Lys = 0.67 and 0.69 in starter and grower, respectively) did not exert any significant effect on FCR from 0 to 21 d and from 21 to 42 d of broilers age, even if a quadratic response was observed in the overall period of trial [45]. When increasing the total dietary Arg:Lys ratio from 1.17 to 2.10 between 21 and 42 d, Fouad et al. [46] did not observe any significant alteration in feed to gain ratios of broilers.

In the present study, broilers receiving the arginine-supplemented diet also showed a significantly higher BW at 33 d while both groups reached the same live-weight at slaughter. It has been reported that the dietary supplementation of arginine from 21 to 42 d (total Arg:Lys ratio = 1.17, 1.40, 1.63 and 2.10) had no effect on BW at processing [46]. Xu et al. [45] observed a quadratic improvement in BW both at 21 and 42 d of age in response to the dietary supplementation of arginine, with the birds fed either a arginine-deficient diet (total Arg:Lys ratio = 0.67 and 0.69 in starter and grower, respectively) or the highest level of arginine supplementation (total Arg:Lys ratio = 2.07 and 2.53 in starter and grower, respectively) showing lower BW compared to the others. Moreover, DFI was similar between CON and ARG group, indicating that the dietary supplementation of arginine did not exert any effect on feeding behavior of broiler chickens at different age. This observation is in accordance with other previous studies [41–46]. However, Corzo et al. [47] reported a significant effect of progressive amounts of dietary arginine from 42 to 56 d of broiler age on feed consumption. Furthermore, mortality rate was similar between the two experimental groups, which is in line with previous findings [41, 46–48]. Based on the results obtained in the present study, the Arg:Lys ratios currently adopted at least in Countries where the animal protein sources are not allowed in feed formulation (i.e. European Union) appears not adequate to exploit productive potential of modern fast-growing broiler chickens.

No significant difference was observed between the groups concerning the incidence and severity of WS, WB and SM. It has been previously reported that the administration of diets with an Arg:Lys ratio of 0.95 and 1.25 exerted no significant effect on the occurrence of WS and WB in 53-day-old broilers [49]. Bodle et al. [17] recently reported that increasing the digestible Arg:Lys ratio from approximately 111–113% to 120–125% reduced the severity of WB while had no effects on WS.

Quality attributes and proximate composition of breast meat were not significantly affected by the arginine supplementation. It has been reported that the dietary supplementation of 153% of digestible arginine in a control diet significantly increased crude protein and dry matter content in breast meat, whereas ash and fat content were significantly improved by supplementing 183% and 168% of digestible arginine, respectively [43]. On the other hand, Fouad et al. [46] observed no significant alterations in the intramuscular fat content of breast muscle of broilers fed diets with different concentrations of arginine (total Arg:Lys ratio = 1.17, 1.40, 1.64 and 2.10) from 21 to 42 d. Considering breast meat quality traits, administering diets with arginine levels from 80 to 140% of NRC recommendation increased L* value and cooking loss, while showed no effects on a* and b* value and drip loss [50]. The dietary supplementation of 0.80, 0.95, 1.10 and 1.25% of *L*-arginine from 42 to 56 d significantly affected lightness (L*) and yellowness (b*) of breast fillets [47]. Finally, also the incidence and severity of foot pad dermatitis exhibited no significant difference in response to the dietary treatment.

Concerning metabolomics, the rPCA models showed differential levels of plasma and muscle metabolites between groups (Figs. 2 and 3, respectively) indicating a clear separation of them according to the dietary supplementation of arginine. In fact, increasing the level of dietary arginine significantly enhanced the plasma concentration of arginine and leucine while reduced that of proline, glutamate, acetoacetate and adenosine. In addition, ARG birds exhibited higher levels of breast muscle arginine and acetate, whereas the concentration of acetone and inosine was reduced. According to these findings, the dietary supplementation of *L*-arginine was able to increase its concentration in both plasma and *Pectoralis major* muscle, indicating that arginine can be effectively absorbed by the intestinal epithelium and can enter the systemic circulation reaching peripheral tissues such as breast muscle. Dietary arginine is absorbed in the small intestine using both sodium-dependent and -independent mechanisms, with the latter showing a greater effectiveness [51, 52]. As most of the arginase activity is located in the kidney [11], a substantial amount of dietary arginine may have passed the brush border and then entered the systemic circulation with only limited degradation. Once in the muscle, arginine could stimulate protein synthesis and cell proliferation [10]. Moreover, plasma concentration of leucine appeared higher in birds receiving the arginine-supplemented diet. Higher plasma levels of leucine have been associated with a greater protein synthesis in skeletal muscle of pigs [53]. Similarly, Baeza et al. [18] reported a positive correlation between *Pectoralis major* weight and plasma histidine concentration, which was numerically higher in ARG birds in this study (4.85 × 10^− 2^ vs. 3.97 × 10^− 2^ mmol/L, *P* < 0.1; data not shown). Taken together, these results indicate that the dietary arginine supplementation may improve anabolic processes within breast muscle probably via protein synthesis enhancement and this merit further in-depth investigations.

Furthermore, the dietary supplementation of arginine appears to modulate energy and protein metabolism. Two ketone bodies, acetoacetate and acetone, showed lower concentrations in ARG plasma and breast muscle, respectively. Ketone bodies can be recruited from blood circulation by peripheral tissues, including breast muscle, and catabolized to produce energy. Therefore, these findings may indicate an increased utilization of ketone bodies in peripheral tissues in response to the dietary arginine supplementation. Fouad et al. [10] reported that dietary arginine supplementation can modulate body fat deposition in chickens. Indeed, Fouad et al. [46] associated the lower abdominal fat deposition in response to the dietary supplementation of arginine to both the increased expression of genes involved in fatty acid β-oxidation and to the reduced expression of fatty acid synthase gene in heart and liver, respectively. A potential effect of the dietary supplementation of arginine on energy and fat metabolism has been previously reported also in meat-type ducks [54].

Glutamate and proline, both of them resulting from arginine metabolism [8, 10], also showed lower concentration in plasma of ARG birds. In mammals, glutamate has been reported to be associated with several physiological aspects such as cell proliferation, biosynthesis of neurotransmitters and other amino acids, immune functionality, acid-base balance and gene expression [55]. Proline is involved in important biological functions related to cellular metabolism, including the regulation of gene transcription and cell differentiation, scavenging oxidants, protein synthesis and structure, cell signaling and bioenergetics [56]. However, in particular metabolic conditions (e.g., nutritional or metabolic stress), glutamate can participate to gluconeogenesis in kidney [55] or enter the citric acid cycle (Krebs cycle) [57]. Similarly, proline metabolism can generate electrons which can enter the mitochondrial electron transport chain to produce ATP [58, 59]. Otherwise, proline can be also degraded to produce α-ketoglutarate, an intermediate of the citric acid (Krebs) cycle [57]. Therefore, it may be hypothesized that the lower concentration of glutamate and proline in plasma of ARG birds may be due to an increased recruitment and utilization of these amino acids in peripheral tissues, possibly the skeletal muscle, to provide energy substrates for the cell.

Inosine represents a metabolite of ATP degradation which can be converted to hypoxanthine and then released into blood circulation [60, 61]. Plasma concentration of hypoxanthine was higher in birds received the dietary supplementation of arginine compared to CON group (6.80 × 10^− 3^ vs. 3.92 × 10^− 3^ mmol/L, *P* < 0.1; data not shown) suggesting that muscle ATP could have been catabolized to provide energy within the cell. Although the molecular mechanism is still unknown, the increased concentration of acetate in breast muscle suggests that the muscle acetate-mevalonate pathway is activated to promote muscle cell development through steroids and/or triterpenoids.

Finally, the adenosine concentration was also reduced in plasma of birds fed the supplemented diet. In mammals, adenosine could be released in plasma by endothelial cells and myocytes in response to ischemia, hypoxia, or oxidative stress [62, 63]. *L*-arginine has also been shown to have marked antioxidant properties [64]. Therefore, the reduction of plasma adenosine might be related to the potential effect of arginine on oxidative status and hypoxic condition likely occurring in breast muscle of fast-growing broiler chickens.

**Fig. 4:**
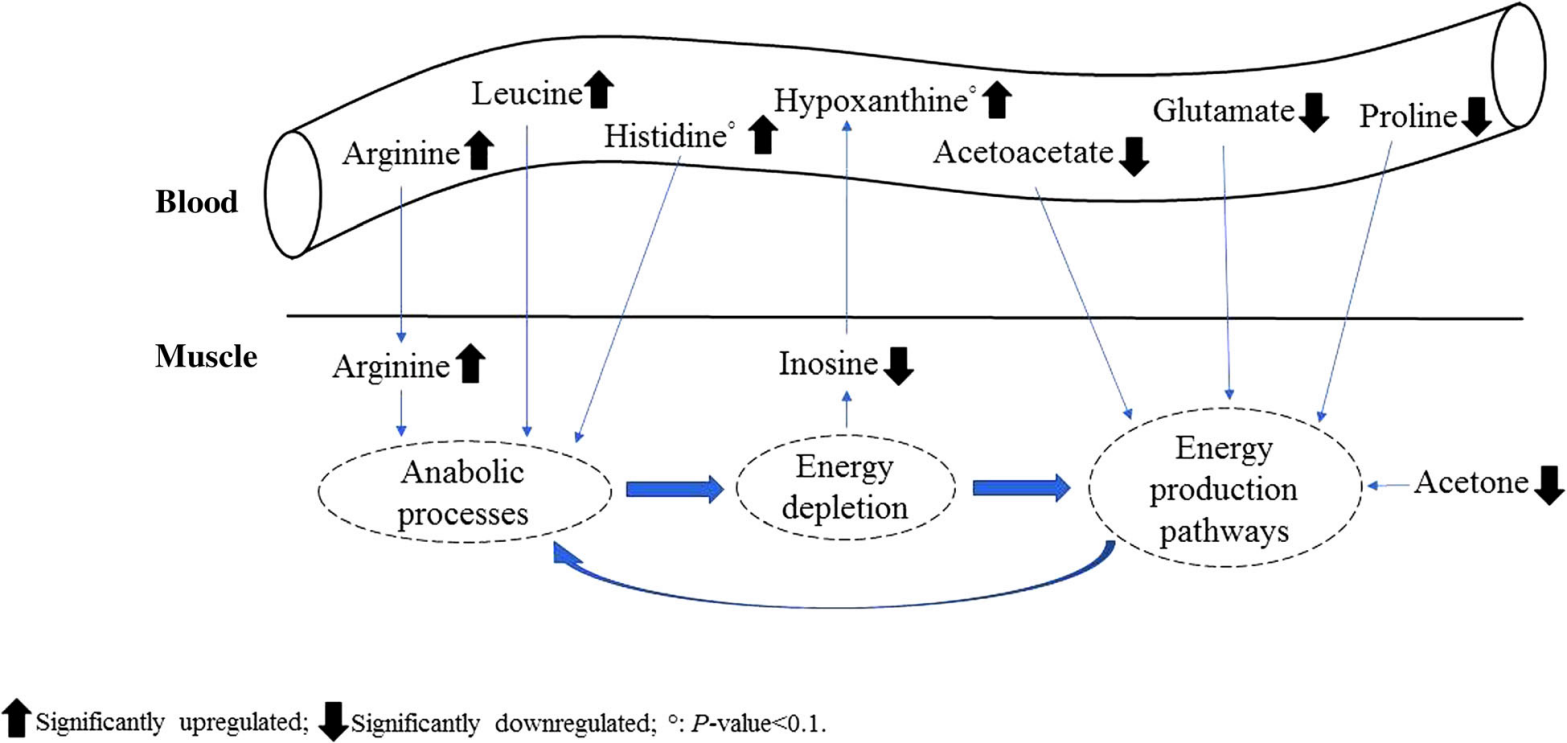
Hypothetical molecular responses to the dietary supplementation of *L*-arginine in broiler chicken

A global hypothesis of the molecular responses to the dietary supplementation of arginine is reported in Fig. 4. Overall, arginine supplementation could stimulate anabolic processes within the muscle and improve feed efficiency. The increased energy depletion, as suggested by the lower value of inosine in breast muscle and the increased concentration of hypoxanthine in plasma, appears consistent with this hypothesis. In turn, skeletal muscle cells may have stimulated the recruitment of several plasma metabolites (e.g. acetoacetate, glutamate, proline) which can be used to restore the energy pool through energy producing pathways (e.g. Krebs cycle).

## Conclusions

Based on our experimental conditions, the Arg:Lys ratios currently adopted at least in countries where the animal protein sources are not allowed in feed formulation (i.e. European Union) appear to be inadequate to exploit the maximum productive potential of modern fast-growing broilers. The Arg:Lys ratios tested herein had positive effects on feed efficiency without showing any negative effect on meat quality attributes, foot pad condition and incidence of breast meat abnormalities. Furthermore, plasma and muscle metabolome showed significant alterations in response to the arginine supplementation. According to this analysis, the improvements observed in feed efficiency in the supplemented group might be likely ascribed to a potential modulatory effect of arginine on energy and protein metabolism and hence on the overall energy homeostasis in broiler chickens. In addition, the present study confirms the usefulness of NMR-based approach in investigating the molecular response to different dietary treatments in avian species. Further studies are warranted to investigate the effects of graded Arg:Lys ratios on productive aspects and meat quality attributes in broiler chickens. In addition, other mechanistic studies are necessary to define and delineate the role of arginine on energy and protein metabolism in breast muscle as well as in other tissues, such as liver and adipose tissue.

## Abbreviations

^1^H-NMR: Proton nuclear magnetic resonance
Arg:Lys: Arginine to lysine
BW: Body weight
DFI: Daily feed intake
DWG: Daily weight gain
FCR: Feed conversion rate
FPD: Foot pad dermatitis
NMR: Nuclear magnetic resonance
NRC: National Research Council
rPCA: Robust principal component analysis
SM: Spaghetti meat
WB: Wooden breast
WS: White striping
